# Sensor-Integrated Microfluidic Approaches for Liquid Biopsies Applications in Early Detection of Cancer

**DOI:** 10.3390/s20051317

**Published:** 2020-02-28

**Authors:** Jessica Sierra, José Marrugo-Ramírez, Romen Rodriguez-Trujillo, Mònica Mir, Josep Samitier

**Affiliations:** 1 Nanobioengineering Group, Institute for Bioengineering of Catalonia (IBEC) Barcelona Institute of Science and Technology (BIST), 12 Baldiri Reixac 15-21, 08028 Barcelona, Spain; jsierra@ibecbarcelona.eu (J.S.); rrodriguezt@ibecbarcelona.eu (R.R.-T.); jsamitier@ibecbarcelona.eu (J.S.); 2 Department of Electronics and Biomedical Engineering, University of Barcelona, Martí i Franquès 1, 08028 Barcelona, Spain; josealfonso.marrugo@icn2.cat; 3 Centro de Investigación Biomédica en Red en Bioingeniería, Biomateriales y Nanomedicina (CIBER-BBN), Monforte de Lemos 3-5, Pabellón 11, 28029 Madrid, Spain

**Keywords:** cancer, circulant tumor DNA (ctDNA), liquid biopsy, microfluidic, sensors, circulant tumor cells (CTC), exosomes

## Abstract

Cancer represents one of the conditions with the most causes of death worldwide. Common methods for its diagnosis are based on tissue biopsies—the extraction of tissue from the primary tumor, which is used for its histological analysis. However, this technique represents a risk for the patient, along with being expensive and time-consuming and so it cannot be frequently used to follow the progress of the disease. Liquid biopsy is a new cancer diagnostic alternative, which allows the analysis of the molecular information of the solid tumors via a body fluid draw. This fluid-based diagnostic method displays relevant advantages, including its minimal invasiveness, lower risk, use as often as required, it can be analyzed with the use of microfluidic-based platforms with low consumption of reagent, and it does not require specialized personnel and expensive equipment for the diagnosis. In recent years, the integration of sensors in microfluidics lab-on-a-chip devices was performed for liquid biopsies applications, granting significant advantages in the separation and detection of circulating tumor nucleic acids (ctNAs), circulating tumor cells (CTCs) and exosomes. The improvements in isolation and detection technologies offer increasingly sensitive and selective equipment’s, and the integration in microfluidic devices provides a better characterization and analysis of these biomarkers. These fully integrated systems will facilitate the generation of fully automatized platforms at low-cost for compact cancer diagnosis systems at an early stage and for the prediction and prognosis of cancer treatment through the biomarkers for personalized tumor analysis.

## 1. Introduction

Uncontrollable cell growth is produced due to the accumulation of genetic and epigenetic modifications that regulate the main tumor cell functions, such as proliferation, survival, development, propagation, and differentiation. This disorder, along with its respective alterations, gives rise to a series of heterogeneous diseases known as cancer. This condition is considered the second cause of death globally and according to the latest estimated cancer statistics available within the Global Cancer Observatory (GLOBOCAN) project, the number of cancer incidences has continued to increase drastically, reaching 18.1 million cases in 2018 and is estimated to be 29.5 million in 2040 [1].

Among several techniques, tissue biopsy is still considered the most common cancer diagnosis procedure worldwide, which requires the removal of a portion belonging to the original lesion. In order to determine a patient-specific tailored treatment, the histologic characterization of the fragment is performed. In many instances, however, the patient that is undergoing tissue biopsy can experience some risks, such as tumour sampling near main organs and vessels, lesions located in tricky regions of the brain etc. Imaging techniques are also used in conjunction with tissue biopsies to offer a better profiling of the tumor. These are, however, inadequate for a thorough study of the tumor, in addition to high radiation levels provided by radiology, which might expose the patient to a health problem. Fortunately, this is now evolving, and the oncological community has intensified its emphasis on radiation safety, contributing to advancements in technology, the implementation of new imaging procedures, and reasonable usage criteria to minimize and limit the exposure to radiation. The detection of minimal residual disease (MRD) is considered a main parameter regarding the patient’s treatment and monitoring, and non-radiation approaches, including magnetic resonance imaging (MRI) scans, are still inconclusive and inadequate [2].

Therapeutic approaches and patient-tailored treatments are generally established based on the tumor molecular profile. The molecular structure of the tumor, however, changes rapidly through time as a result of a combination of internal and external factors with numerous consequences. The tumor has temporal and spatial heterogeneity [3] and is produced in some cases by stress over certain tumor regions that alter the genomic structure of it [4]. Even inter-and intratumoral convergent phenotypic evolution has been discovered in several regions of the same tumor [5]. The challenge of conducting a single biopsy-based therapeutic intervention and monitoring underestimates the magnitude of the tumor genomics, which is highlighted by its heterogeneity. Moreover, for a more effective tailored diagnosis of cancer, the progression of the tumor must be tracked at several time intervals. Several tissue biopsies, from both primary and metastatic lesion, are taken as the steps to be considered. However, the inherent health hazards, possible surgical complications, and economic considerations are just some of the many pitfalls in the acquisition of tissue biopsies, underlining the pointlessness of performing several biopsies. However, in certain body locations, the threat of metastatic proliferation or cancer “seeding” can be raised by the removal of certain tumors that are unable to be reached through biopsy [6].

As a result, it is important to search for minimally invasive biomarkers so that an early detection technology can be available, with its respective frequent monitoring along with cancer therapy.

There has been unprecedented eagerness in the medical community to explore the molecular environment of solid tumors by a blood draw, a process known as Liquid Biopsy. Liquid biopsy has very relevant advantages, including its minimal invasiveness, lower risk, and it has the potential of being analyzed with the use of lab-on-a–chip (LOC)-based platforms with low consumption of reagents, and does not require specialized personnel and expensive equipment for the diagnosis. Several cancer-associated parameters such as the tumour’s molecular landscape and MRD among others could be determined by the application of liquid biopsy, which has demonstrated an enormous and valuable potential [7,8]. Lately, liquid biopsies have also gained interest for early diagnosis of tumors, therapeutic guidance, and recurrence monitoring [9].

Liquid biopsy has its origin in the fact that the body fluids contain biomaterials that are originated from different tissues, including cancerous ones. Undeniably, due to the fast evolution of cancer cells, tumor-derived nucleic acids and vesicles can be constantly released into the circulation, and even the whole tumor cells are separated from the primary tumor and enter the blood circulation, which explains the epithelial-mesenchymal transition [10,11]. Thus, detecting and characterizing circulating tumor DNA (ctDNA), exosomes and circulating tumor cells (CTCs), enable clinicians to cross-examine the evolution of different human cancers in a non-invasive way. There is a growing literature that documents how liquid biopsies are informative for detecting and characterizing CTCs [12,13,14,15,16,17,18], ctDNA [12,15,16,17,18,19,20,21,22,23,24,25,26] and exosomes or extracellular vesicles [20,27,28]. The information extracted from these biological components allows for early cancer detection and real-time monitoring of therapies; identification of therapeutic targets and resistance mechanisms; along with understanding and characterizing of metastasis mechanisms, metastatic relapse or metastatic progression in cancer patients [29,30,31].

In the last years, microfluidic platforms, integrating biosensors such as LOCs, have been getting more interest from the research community as an excellent tool to analyze liquid biopsy. This technology offers several advantages for diagnostics, as it can improve efficiency, portability, analysis speed, automation, and miniaturization, as well as lower the consumption of a reagent, lower cost and make usage easier. Apart from blood, other corporal fluids (saliva, urine, cerebrospinal fluid (CSF), among others) may contain tumor-associated material. Therefore, liquid biopsies will certainly expand in the future as a key diagnostic element for early detection of cancer.

## 2. Lab-on-a-Chip Platforms for Liquid Biopsies Applications

### 2.1. Platforms Based on Circulating Tumor Cells Analysis

**Figure 1:**
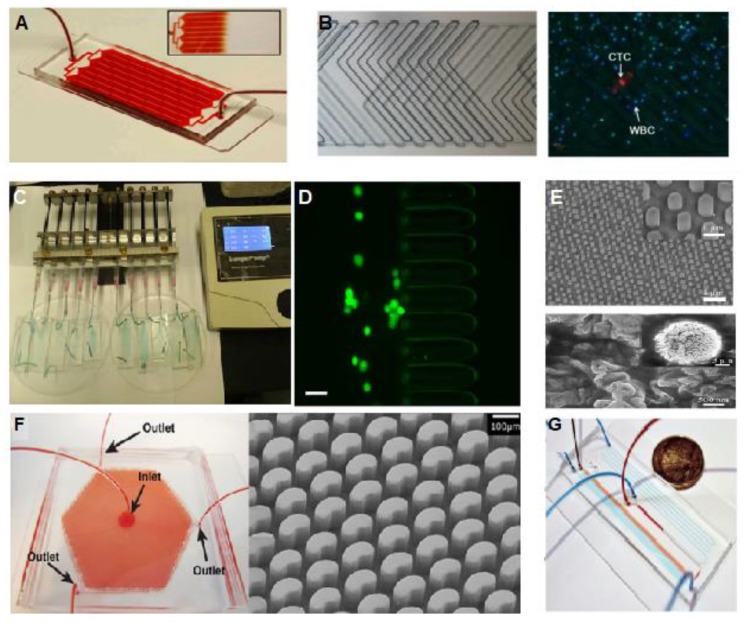
(**A**) The HB-Chip is composed by a collection of microfluidic channels with a single fluid entrance to multiple channels to move again to a single channel to exit, which allows uniform blood flow inside the device [35]. Copyright 2010 National Academy of Sciences. (**B**) Herringbone chip with microstructures in a double-sided channel. The chip was validated with real blood samples from lung cancer detection. CTCs were capture and stained inside the chip with antibodies against CK in red, DAPI in blue, and CD45 in green [36]. “Republished with permission of [Royal Society of Chemistry]; permission conveyed through Copyright Clearance Center, Inc. (**C**) Microfluidic platform for highly efficient retention of CTCs based on a physical variation on cell deformation and diameter. The chip is composed by micro-ellipse filters which were able to detect CTCs from the detection of colon, breast and non-small-cell lung (NSCLC) cancer [38]; Copyright © 2017, Springer Nature. (**D**) Tumor cells captured by a microfluidic platform for high capture efficiency of CTCs (Green), where the CTCs pass through the ellipse filters (Scale bar = 50 µm) [38]; Copyright © 2017, Springer Nature. (**E**) Nanopillar arrays with a diameter of 650 nm with cell membrane details of the capture cells into the device [39]; Copyright © 2017, Springer Nature. (**F**) OncoBean Chip with micropost structures inside the device. The device allows the cell separation by affinity at high flow rates, applying a radial flow, which produces a variation of the shear profile across the device. Licensed under CC BY-NC International License. The final, published version of this article is available at [41]. (**G**) Microfluidic device based on the separation of CTCs clusters by Size and Asymmetry. The device was tested with whole blood (red) and colored PBS buffer (blue) [42]; Copyright © 2017, Springer Nature.

Since CTCs were discovered a century ago, their detection in liquid biopsies has been attracting a lot of interest as a promising tool for cancer diagnosis, and the use of microfluidic technologies for this purpose has now gained importance. Microfluidic devices focused on CTCs analysis include cellular segregation and examination, demonstrating high efficiency, reactivity and selectivity; low sample amount and reagent costs; and high liquid control capacity [32]. Most of the studies showed the development of microfluidic chips for CTCs capture using the affinity towards a specific cell membrane protein called “Epithelial Cell Adhesion Molecule” (EpCAM) [33]. This capture is achieved by the immobilization in the channels of the microfluidic system of specific antibodies against this EpCAM protein. Nevertheless, microfluidic systems using this method have a restricted capture capacity due to the small available area for ligating antibodies in a conventional microfluidic channel. In order to overcome these limitations, improved microfluidic set-ups were ideated to provide an increased interaction surface. With this purpose, Herringbone shape array [34,35,36,37], micro-post array [38,39], and nanopillar array [40] were reported. These strategies are based on a design of the microfluidic channel with a high surface/volume ratio, compared with the traditional channel patterns, increasing in this way the efficiency of the retention of CTCs (Figure 1).

Apart from functionalizing the microfluidic channel with immunoreceptors, other strategies were focused on the modification of immunomagnetic beads with the same EpCAM antibodies, which flowed inside microfluidic channels, such as CellSearch^®^. This platform is the standard method for CTC enrichment and is the only blood test accepted by the Food and Drug Administration (FDA) for detecting CTCs in patients with metastatic breast, prostate, and colorectal cancer [43,44].

The method proposed by GILUPI Company explores a different technique for CTC separation. The GILUPI CellCollector^®^ avoids the use of active pumping, since the flow of body fluid is used for this purpose. Thus, it has no restrictions on limited blood volume like the traditional CTC isolation methods. This technology was designed to collect with a functionalized probe with antibodies against EpCAM targeted cells in vivo into the vascular system, where the total volume of blood can be analyzed, increasing the chance of CTCs isolation [41].

Previously described devices have proved to be very sensitive in certain types of cancer and under certain conditions. However, EpCAM antibody is only specific for a few types of tumor cells, and considering the high heterogeneity of cancer, it cannot be used as a universal biomarker. To overcome this problem, other strategies have been developed based on negative selection, where blood cells are immobilized on the microfluidic chip surface and so the rest of the cells, mostly CTCs, are recovered at the chip’s output [45].

**Figure 2:**
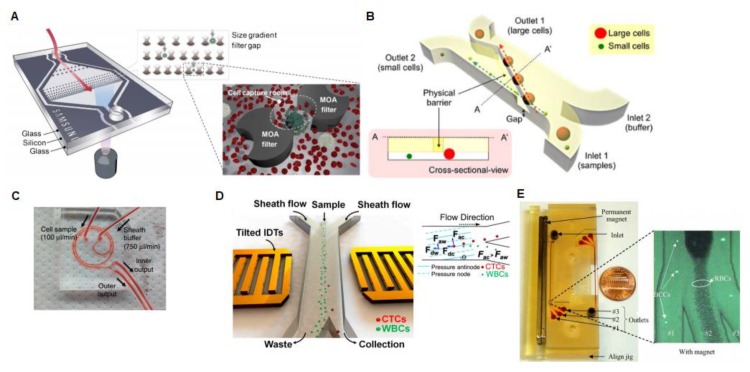
(**A**) Multi-obstacle architecture (MOA) size-gradient filter chip. For separation and cell clogging purposes, two different filters are integrated into the device respectively, the second one being mitigated by a “cell capture area” in-between [46]. Republished with permission of [Royal Society of Chemistry]; permission conveyed through Copyright Clearance Center, Inc. (**B**) The Microfluidic Cell Sorter (μFCS) is composed of a fluidic channel with two inlets and two outlets. In the microchannel, a gate is included diagonally for separating cells by their sizes. Thus, the small cells pass through the hole and are collected in outlet 2 by laminar flow. Meanwhile, the larger cancer cells pass through the barrier and are collected at another outlet [47]. Licensed under CC BY-NC International License. The final, published version of this article is available at [48]. (**C**) This system is made of two-loop spirals with two inlets and outlets with a radius of 10 mm, approximately. The microchannel width is 500 μm and outlet bifurcation is designed to be optimum between 150 and 350 μm. The device separates the cells based on their size using hydrodynamic forces [53]. Reprinted by permission from Springer Nature Customer Service Centre GmbH: on behalf of Cancer Research UK: [Nature Protocols], Copyright (2015) (doi:10.1038/nprot.2016.003). (**D**) Schematic of the working principle of tilted angle standing surface acoustic waves (taSSAW) microfluidic device for cancer cell isolation. In this platform, the larger CTCs experience a bigger acoustic radiation force (Fac) than WBCs (Faw). Consequently, CTCs suffer a greater vertical displacement than WBCs. Fdc and Fdw represent the drag force experienced by CTCs and WBCs. Copyright (2015) National Academy of Sciences [54]. (**E**) Continuous paramagnetic capture (PMC) microseparator device, where fluorescent breast cancer cell lines (BCCs) pass through the microchannel at a flow velocity of around 0.05 mm/s with an external magnetic alteration of 0.2 T. The separation is achieved based on the native magnetic properties of cells and can separate 94.8% of breast cancer cells. Reprinted with the permission of the publisher (Taylor & Francis Ltd.) [56].

Moving from the immuno-entrapment methods, other methods have been proposed based on the differences in physical features between CTCs and normal blood cells. With this purpose, microfluidic technologies were developed to sort CTCs based on their different cell size [46,47,48,49,50], density [51,52], compressibility [53,54], and native magnetic properties with regards to blood cells [55] (Figure 2).

**Figure 3:**
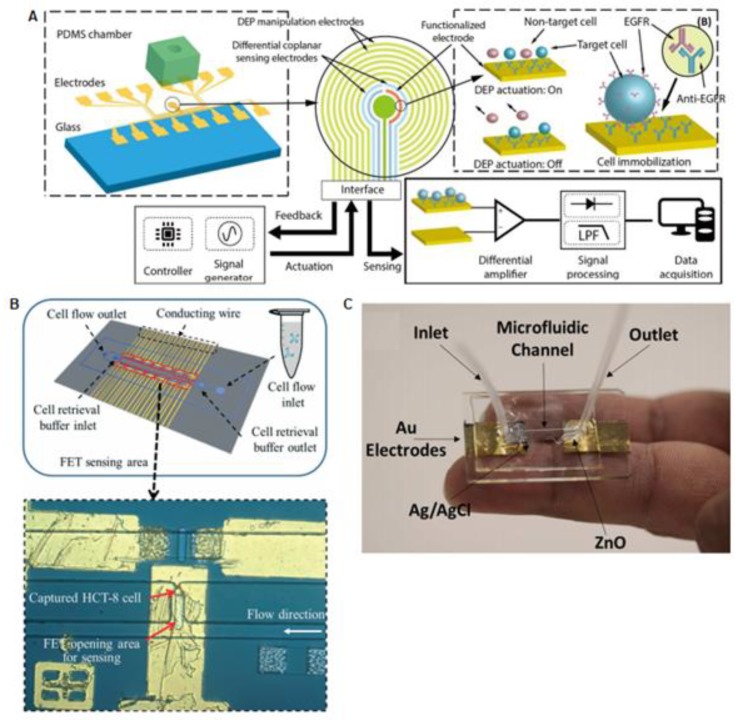
(**A**) Diagram of dielectrophoresis microfluidic platform composed by a chamber made of PDMS and a capacitive sensor for circulating tumor cell detection. Thus, tumor cells are separated by DEP and a stepping electric field [65]; Copyright © 2018. The Korean BioChip Society and Springer-Verlag GmbH Germany, part of Springer Nature. (**B**) Integrated microfluidic platform is composed of a microfluidic device and a collection of FET-sensors inserted in epoxy substrate, able to capture cancer cells on the transistor sensing surface [59]; republished with permission of [Royal Society of Chemistry]; permission conveyed through Copyright Clearance Center, Inc. (**C**) Microfluidic pH Sensor based on the measurements between a silver–silver chloride and zinc oxide electrodes for CTCs recognition in blood. The device detects the cancer cells based on changes of pH in the extracellular environment [64]; Copyright © 2017, American Chemical Society.

In general, the CTC separation methods described so far have in common the absence of integrated detection of the isolated tumor cells into the microdevice. Most of the systems, to elucidate the presence of the CTCs, use fluorescent-labeled antibodies specifically attached to the CTC, and the fluorescence label is detected with an external microscope. However, some authors have gone one step further and have combined microfluidic isolation techniques with integrated sensors for CTCs analysis in situ. Figure 3A shows one example of a microfluidic device with integrated read out for CTCs detection based on a field effect transistor (FET), using the high transconductance gain of CTCs [56]. Compared to conventional external fluorescence CTCs detection devices, integrated electrical sensing is compact, portable, sensitive, and automatized. Based on this principle, Yi-Hong Chen and their colleagues developed a microfluidic device containing a CTC-specific aptamer ligand on a FET surface, which could perform detection and isolation of CTCs. The device is composed of a dual-layer with two inlets and 14 individual trapping chambers. The chip was tested with human colon cancer cell lines (HCT-8), as a CTC model and blood samples spiked with HCT-8 cells. The device was able to capture a maximum of 42 from a total of 1000 cancer cells [57].

In a similar FET configuration, Pulikkathodia et al. reported a high electron mobility transistor (HEMT), a multiplexed sensor integrated into the channels of a chip to detect colorectal cancer cells (HTC-8), as is described in Figure 3B. The results revealed high sensitivity that can reach a single-cell resolution in a short time of 5–10 min [58].

Other electronic approaches have explored the junction of impedance sensing and dielectrophoresis (DEP) method in a microfluidic chip. Previous studies revealed that the use of impedance spectroscopy is an excellent tool for label-free characterization of cells, which provides information about electrical cell parameters [59]. Nguyen and Jen presented a microfluidic device with circular electrodes and a single microfluidic channel, see Figure 3. This device was tested with A549 lung CTCs and blood samples (RBCs to CTCs ratio of 13), being able to discern between the two cell populations due to their different resistivity [60].

Moving from electronic to optically read out, some methods like internal reflectance spectroscopy, surface plasmon resonance, and evanescent wave sensing, among others, represent excellent methods for being integrated into microfluidic devices because they are label-free. Kumeria et al. described a microfluidic nanopore reflectometric interference spectroscopy (RIfS) device composed of microchannels in Anodic Aluminum Oxide (AAO) substrate modified with anti-EpCAM for detecting CTCs. The binding of CTCs to the antibody-modified AAO surface originated a wavelength shift in the Fabry-Perot interference fringes as a principle of detection [61], see Figure 3.

In the work proposed by Tzu-Keng Chiu et al. separation and cell analysis were performed also with optical techniques. The microfluidics-integrated separation method performed with optically induced dielectrophoresis (ODEP), a method based on cell separation by size, isolates CTC from the blood cancer patients with high purity. They designed the device for complex cell manipulation steps by ODEP, such as suspension, transportation, collection, and purification of the harvested cancer cell. The simplicity of the microfluidics with one main channel, contrast with the complexity of the dynamic square light image array to manipulate the cells. The device was tested with 8 mL of blood sample and 6.25 H209 cancer cell clusters per milliliter, with a result of excellent purification of the cell with a rate of recovery of 91.5% ± 5.6% and 70.5% ± 5.2%, respectively [62].

Recently, researchers have shown an increased interest in developing bioreceptor-free integrated analysis methods by indirect CTC identification. The study performed by Tzu-Keng Chiu et al. resulted in a bioreceptor-free CTC detection platform based on the optical read out. This platform uses the metabolic performance of cancer cells, such as the production of lactic acid by CTCs, to indirectly quantify these cells. The proposed device can perform a continuous cell-counting by the formation of a micro-droplet, to end with a cell single detection with optical transduction of lactic acid, obtaining the determination of a quantitative cell inside the micro-droplets. It is important to highlight that the response was very selective and does not detect the presence of similar cells such as leukocytes [63].

Another indirect method of cancer cell detection is the one based on pH monitoring, as shown in Figure 3C. In this case, the metabolic change produced by the CTC was a reduction on the surrounding pH. pH-sensing studies were done by using potentiometric methods with an Ag/AgCl reference electrode and a ZnO working electrode. The in vitro evaluation in a microfluidic cell was performed for determining its capability to differentiate three cell lines (A549, A7r5 & MDCK), which was demonstrated based on their difference in pH values. Nevertheless, the proposed device was not still tested with blood samples [64].

### 2.2. Platforms Based on Exosomes Analysis

Extracellular vesicles (EVs), known as exosomes, are particles detached from cells composed of a lipid bilayer with a diameter from 40 to 200 nm and containing nucleic acid, proteins, and metabolites from the native cell [65,66]. Pan and Johnstone discovered exosomes in 1983 when culturing sheep reticulocytes at McGill University [67]. Recent studies have shown that EVs are involved in several physiological processes, including intercellular communication, tissue repairing, and immunologic response regulation [68,69]. The angiogenesis and evolution of tumors, physiological cancer-related processes, are also strongly influenced by exosomes [68,69,70]. Due to the increasing attention that is been received, exosomes can be considered a potential biomarker for early cancer diagnosis, giving their valuable and quantifiable information about the primary and metastatic lesions that are present in body fluids, such as plasma, urine, or saliva, which permits its minimally invasive monitoring.

However, the most relevant feature of this molecule, comparing with the other liquid biopsy biomarkers, is that exosomes are secreted at the early stage of the tumor cells, and therefore, it is an excellent hallmark for early detection of cancer [71]. Nevertheless, the strain of the extraction procedure is one of the major factors preventing the identification and use of exosome biomarkers. Among several approaches, the most commonly used method for the isolation of extracellular vesicles is ultracentrifugation. Nonetheless, factors such as its long processing time (4–5 h), considerably poor yield (5%–25% recovery) and high maintenance costs makes it very inconvenient and impractical. Commercially available precipitation kits are simple to use without the use of trained personnel or facilities. However, the protocol needs overnight incubation and cross-contamination, as well as lower purity of the acquired exosomes is obtained compared to other methods. Microfiltration and other size exclusion techniques are low cost and simple methods, but present issues regarding pore clogging, shear stress-induced damage, and analyte loss [72]. Immunoaffinity methods can be used in shorter periods (2–4 h) in order to purify a specific exosome population expressing a specific surface marker; nevertheless, in cost and complexity terms, it is not a better approach. Common exosome markers, such as CD9, CD63, and CD81 [73,74], are being used for the non-specific exosome capture, either functionalized onto a channel surface or onto magnetic beads, as seen for CTCs entrapment systems, whereas cancer-associated exosomes can also be specifically secluded using EpCAM [75,76], CD24 [77], and CD37 [78] among others.

There are commercially available microfluidic systems that just incorporated the separation and extraction of the exosomes, but the readout is performed externally, such as the ExoChip [65], nPLEX [79], iMER [80], ExoSearch [81], Nano-IMEX [82], and µMED [83]. Besides, other microfluidic systems were explored based on size and other intrinsic properties [84,85,86,87,88,89,90,91].

Regarding the external detection of exosomes, molecular counting-based approaches are considered the main techniques, including dynamic light scattering [92]; conventional antibody-based immunoassays, like flow cytometry [93]; and optical methods [94,95], including Surface Plasmon Resonance (SPR). However, even with their superior advantages, some of these methods are limited by several aforementioned factors regarding time consumption, expensive equipment, and sample handling, among others [96]. All these methods were described and compared in several review articles [97,98].

However, also, integrated sensing systems in exosomes extraction microfluidic chips have been reported. Regarding the analysis of biomarkers related with exosomes on-chip, several benefits over the existing established protocols have been presented, including selectivity, sensitivity, portability, cost, and automation. Some analytical methods integrated in the microfluidic chip are based on colorimetric detection of surface proteins analysis [99], immunoelectrophoresis [100], qPCR [101], on-chip ELISA detection [102], and mass quantitation [103], among others.

**Figure 4:**
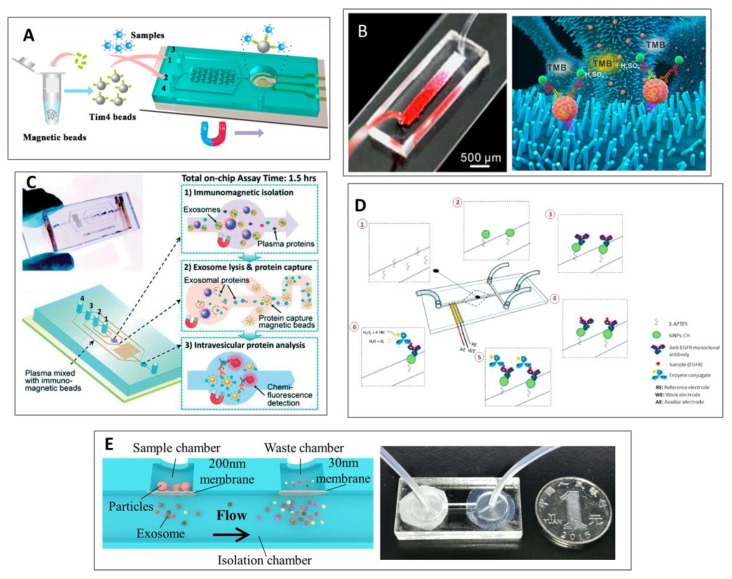
(**A**) Scheme of ExoPCD-chip; a microfluidic platform for isolation and detection of exosomes. Reprinted with permission from [104]. Copyright (2018) American Chemical Society. (**B**) The red ink stuffed into the ZnO nanowires chip with colorimetric analysis for exosomes detection. Reprinted with permission from [40,105]. Copyright (2018), Elsevier. (**C**) Integrated microfluidic chip with specific immunomagnetic isolation and protein analysis of exosomes. Licensed under CC BY-NC 3.0 Unported License. The final, published version of this article is available at [107]. (**D**) Scheme of the channel modification and the antibody interaction in the electrochemical integrated sensor. Reprinted with permission from [106]. Copyright (2019), Elsevier. (**E**) (left) On-chip isolation and detection of urine-derived exosomes using a double-filtration approach. (right) Image of the assembled integrated microfluidic chip. Licensed under CC BY-NC 4.0 International License. The final, published version of this article is available at [110].

Xu et al. presented the ExoPCD-chip (Figure 4A), a microfluidic platform, consisting of two essential approaches: (1) on-chip isolation and enrichment, and (2) in-situ electrochemical characterization from blood-related exosome samples. The microchip is designed with a set of Y-shaped micropillars and an Indium Tin Oxide (ITO) electrode pattern. The channel with micropillars is made of polydimethylsiloxane (PDMS) and is made to efficiently mix the fluid so that it increases the collisions between the exosomes and the antibody-conjugated magnetic beads. Employing a magnetic enrichment, these captured exosomes can be subsequently analyzed on the electrode’s surface [104]. Additionally, it can also trap tumor-derived exosomes with a considerably rapid response (3.5 h), using little sample (30 μL). Besides, they used the system with real clinical samples from hepatic carcinoma patients and obtained a clear correlation between exosomes and tumorigenesis [104].

Another microfluidic design, in which there is an integration of several steps, was presented by Chen et al. [105]. It consisted of a 3D macroporous chip device with integrated ZnO nanowires to effectively isolate exosomes, as well as quantify them by colorimetric assay (Figure 4B). The ZnO nanowires together with the 3D interconnected macropores confer a great surface area as well as chaotic mixing, increasing the efficiency to capture the exosomes [105].

A microfluidic electrochemical immunosensor was designed by Ortega et al. for the identification of Epidermal Growth Factor Receptor (EGFR) in exosomes from breast cancer patients, by employing a platform made of silica nanoparticles coated with chitosan (SiNPs-CH) (Figure 4D) [106]. This device could capture the exosomes by coating the central channel with anti-EGFR antibodies on the surface of the SiNPs-CH. The microfluidic immunosensor presented high stability, selectivity and sensitivity due to the use of monoclonal antibodies.

Another exosome isolation and detection study was done by He et al. [107]. They produced a microfluidic device for isolation of circulating exosomes using a specific immunoassay with magnetic microparticles and further protein detection (Figure 4C) [50]. Compared to the other surface-based approaches, this immunomagnetic system permits higher throughput and increased sensitivity [108]. The PDMS chip uses a cascading microchannel circuit with different sequential steps to drive the exosome first to isolation and enrichment, and then to be chemically lysed for precipitation with antibodies, and finally detected with a fluorescent-labelled secondary antibody [109].

As mentioned before, blood is not the only body fluid in which this type of biomarker can be found. Liang et al. [110] developed a double-filtration approach in order to effectively isolate, enrich and quantify urine-derived exosomes from patients suffering from bladder cancer (Figure 4E). Exosomes ranging from 30 to 200 nm were size-isolated and enriched by means of two polycarbonate membranes. Subsequent analysis of the captured exosomes was done using on-chip ELISA, which considerably simplified the chip testing [110]. Their results strongly suggested that patients suffering from bladder cancer had a significantly higher concentration of urine-derived exosome than healthy controls.

### 2.3. Platforms Based on Circulant Tumor Nucleic Acids Analysis

Nucleic acid (NA) biomarkers, such as cell-free DNA (cfDNA), circulating tumor DNA (ctDNA), messenger RNA (mRNA), and micro RNA (miRNA), can be biological properties exploited in diagnostic applications and for cancer monitoring. The quantification of ctDNA could be a powerful parameter for early detection, progression and prognosis of tumors [54].

Cell-free DNA (cfDNA) is defined by Peters et al. [111] as the fraction of extracellular DNA that is not associated with any cell structure. ctDNA is considered as a biomarker because of its presence in body fluids, and its relation to several pathological and physiological mechanisms, such as cancer, prenatal diagnosis, coagulation, transplants, and cardiovascular diseases, among others. A small portion of cfDNA originated from cancer cells is known as ctDNA [112], and it might present the same genetic mutations as those occurring in the primary tumor [2]. Consequently, ctDNA could represent an alternative way for a non-invasive and free solid biopsy for cancer prognosis and evaluation. Normally, necrotic and apoptotic cell debris are eliminated from the tissue by a physiological process that involves the infiltrated phagocytes. The combination of this process with accelerated cell renewal, which is not so effective in cancer cells, leads to an increase of necrotic and apoptotic cell debris. As a result, the ctDNA within apoptotic and necrotic cells is discharged into the bloodstream [4]. The main technologies used in the laboratories for ctDNA profiling are quantitative reverse transcription polymerase chain reaction (qRT-PCR), microarray hybridization, and next-generation sequencing (NGS) [90]. Currently, available technologies cannot perform the identification and profile of these biomarkers with precision, in an easy way, with low cost devices, and with high throughput analysis.

Moreover, the common laboratory techniques for DNA purification and detection, as well as the different laboratory methods used, frequently display different results, which could augment the range of the standard deviation [113]. The reasons for these alterations involved analyte loss because of the extraction method, nucleic acid degradation due to long assay times, and distinctive PCR amplification rates, among others [90].

Some more sensitive techniques have been applied to detect exosomal DNA, including surface-enhanced Raman scattering (SERS) [114], electrochemical biosensors [115], and droplet digital PCR (dPCR) [116]. Nonetheless, all these approaches depend on DNA extraction, which results in variable DNA yield, sequence bias, sample contaminations, and DNA degradation [117].

Currently, new technologies have been proposed for improving the sample treatment and DNA analysis at the nanoscale, in order to overcome the limitations of the previously described techniques. These technologies involved methods based on free-solution electrophoretic detection for increasing the variation in electrophoretic mobility of the target-probe, with drag-tags and single-stranded DNA binding protein [118], isothermal amplification inside hydrogel microparticles to discriminate different reactions [119], and other biosensor-based approaches reported [120].

**Figure 5:**
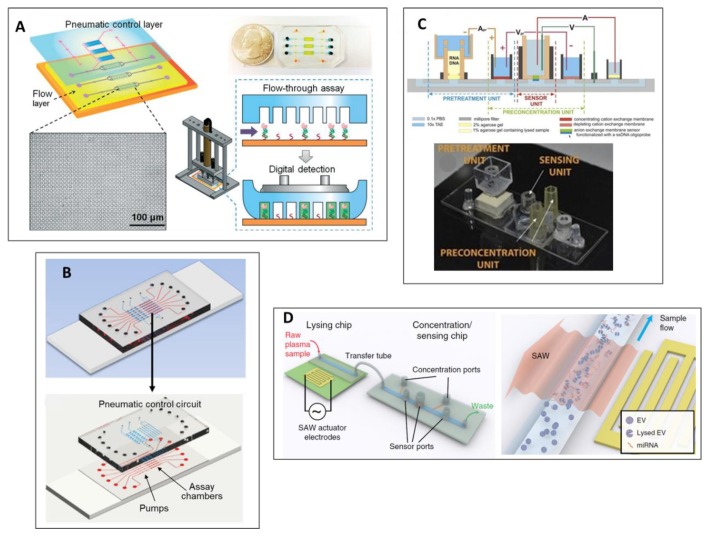
(**A**) Microwell-patterned microfluidic analysis chip. Reprinted with permission from [121]. Copyright (2018), Royal Society of Chemistry. (**B**) Schematic illustration of the assembled chip of the MERCA system for nucleic acid detection. The microfluidic chamber is functionalized with probes to capture target miRNAs; (bottom) design of the MERCA chip. The three-layer PDMS/glass chip integrates seven units working parallelly, consisting of an assay chamber with a three-valve pump. Reprinted with permission from [123]. Copyright (2019), Science Direct. (**C**) Microbiochip integrating a unit for nucleic acid extraction, preconcentration and sensing. Reprinted with permission from [124]. Copyright (2015), Elsevier. (**D**) Principle of detection and chemical-free lysis of exosomes for an integrated microfluidic platform for miRNA analysis. Licensed under CC BY-NC 4.0 International License.

Zhang et al. [121] described an approach to effectively isolate and detect mRNA, which consisted of a microwell-based microfluidic platform that integrates two different steps; first, the target capture and tagging and then a PCR-free mRNA detection (Figure 5A). A pneumatic control system was designed to optimize the workflow and to reliably monitor the flow rates and binding kinetics, which had an enormous impact on the reproducibility of the test [121,122].

A highly sensitive, miRNA study, using an integrated Microfluidic Exponential Rolling Circle Amplification (MERCA) system, was reported by Cao et al., 2019 [123] (Figure 5B). As a proof of concept, different biological samples were analyzed in MERCA system.

Another integrated microfluidic technology was based on an ionic membrane modular biochip platform developed by Slouka et al., 2015 [124] (Figure 5C). With this modern technique, it is possible to rapidly measure (30–60 min) a maximum of three different miRNAs, stating an LOD of 106 copies with high selectivity and sensitivity, using raw cell media or cell lysate as the substrate. This biochip incorporates three different modules: (1) NA extraction; (2) cell waste, protein and long RNA separation from short miRNAs; and (3) specific miRNAs capture. An enrichment unit of the concentration NA is used that not only decreases the test time, but also enhances the system’s tolerance. The final target extraction is carried out by a probe-functioned membrane that enhances selectivity and allows the effective quantification of the target probe. [124].

An innovative chemical-free exosome lysing system, coupled with membrane sensing, was integrated and presented by Ramshani et al. [125]. The purpose of this study was to develop a PCR-free diagnostic platform in order to quantify, in a one-step manner, exosome-derived miRNA from untreated plasma samples (Figure 5D). This microfluidic platform was specifically designed to fulfill three different purposes: (1) efficiently lyse the exosomes, (2) NA enrichment for subsequent analysis, and (3) quantification of target miRNA. The chemical-free lysing method is based on an integrated surface acoustic wave (SAW) chip, which physically lyses exosomes. An enrichment and sensing chip was then employed, based on two sets of ion-exchange membrane [71]. The chip’s detection principle relies on ion-exchange membranes, where the anionic one is functionalized with a complementary probe. The current-voltage interaction throughout the membrane sensor is drastically shifted during hybridization, which can be directly linked to miRNA levels in the sample [125].

## 3. Conclusions and Future Trends

Liquid biopsy has recently appeared as a novel technique for early diagnosis of cancer and is considered one of the most promising technologies for monitoring the evolution of the disease during treatment. However, the ability to detect, characterize and quantify ctDNA, exosomes and CTCs represents a remarkable challenge due to their very small concentration in blood. In the past years, the development of microfluidic systems for isolating and detecting body fluid cancer biomarkers has been improving by integrating sensing approaches in LOCs, which provide several advantages compared to the traditional methods like better efficiency, portability, time of analysis, automation and miniaturization, as well as lower consumption of reagents, time and cost effective and easier usage. This review shows the most relevant reported integrated methods for liquid biopsy separation and analysis. Some of the published works have presented high sensitivity that offers up to single-cell resolution, as well as the possibility to study the cell interaction, which is not possible with traditional microfluidic devices. The integration of electronic/electrochemical/optical sensors in microfluidic devices represents an excellent approach for identifying cancer liquid biomarkers avoiding additional labelling and external extra-steps. Moreover, these platforms have opened new avenues for quantifying CTCs through other biological features such as metabolic cell activity. Thus, the development of this kind of integrated platform provides new insights for more sensitive and effective liquid biopsy devices that allow for the consideration of other kinds of biological and physical cell properties.

Nevertheless, most of the reported devices are in an early development stage and were not tested with a blood sample from a cancer patient. Exploiting the effectiveness of these systems in real patient samples could provide a clear landscape of the tumor heterogeneity, knowing better the aggressiveness and the overall molecular scenario, with more accuracy compared to traditional systems. In fact, the majority of CTC and exosomes separation based on immuno-separation just consider one type of antibody, the EpCAM. However, the tumors have a rich heterogenic mixture of different cells with distinct morphological and phenotypic profiles, which brings different gene expression, motility, proliferation, metabolism, and metastatic potential, and therefore, a different and individual treatment. The immuno-platforms based on positive capture of CTCs and exosomes need to be included in future multiparametric antibodies for considering the entrapment of the rest of the cells contained in the tumor. In contrast, the genomic analysis thought ctNA already considered a different type of gene expression, these platforms being closer to full incorporation as real liquid biopsy analysis tools in Hospitals.

Exosome analysis, along with other tumor materials, such as CTCs and ctNA, are considered the foundation of emerging technologies for liquid biopsy applications, which are only beginning to be studied in order to be used for early diagnosis and personalized medicine. The development of these technologies allows low risk and low-cost testing of samples and plays a crucial role by offering an enormous opportunity for microfluidic-based solutions in routine early detection of cancer. It is interesting that several developments illustrated here have taken place over the past decade, suggesting that this area is still in its early stages. This compilation of literature provides a basis for potential advances by introducing new methods to collect, identify and analyze cancer-related biomarkers. These developments also provide guidance to further work needed to implement the vision of point-of-care applications based on these platforms.
